# What's the true costs of wounds faced by different healthcare systems around the world?

**DOI:** 10.1111/iwj.14491

**Published:** 2023-11-14

**Authors:** Douglas Queen, Keith Harding

**Affiliations:** ^1^ Editor, IWJ; ^2^ Editor‐in‐Chief, IWJ

For many wound carers globally getting a handle on the costs associated with the management of chronic wounds is difficult, if not impossible. There are some published figures geographically but most of these are not ‘standardised’ to permit comparison easily or directly. One consistent theme from a number of these studies, however,^1^, ^2^, ^3^, ^4^, ^5^, ^6^, ^7^, ^8^, ^9^, ^10^, ^11^, ^12^ is that they relate the costs of wounds, extrapolated or otherwise, to the total geographic healthcare costs. This provides a percentage figure as a benchmark.

While most of these studies capture or measure their wound care costs differently making a direct comparison between geographies difficult, they at least provide some measure to permit a comparison. Another weakness of any comparison between studies or geographies is the time frame of measurement. Indeed, a simple literature search shows both the paucity of data generally, in some geographies and the outdatedness of the data in others. Cost studies are tedious, difficult, lengthy and expensive, as such they are seldom carried out.

Due to the difficulties of capturing such cost data most of these studies caution their results as an underestimate of the costs involved.^13^ With that in mind the *IWJ* editors decided to provide an annual appraisal of the likely wound care costs by geographical area around the world.

The data presented in Table 1 provides an estimate of the likely costs of wounds within most of the countries of the world. This provides an international picture of the possible costs of wounds. We will endeavour to update this annually to provide more up‐to‐date figures for those interested.

**TABLE 1:** Estimated wound care costs by country.

Country	Estimated spend on wound care in 2019 (PPP International $ Billion)
United States	$126.8646
China	$26.9452
Japan	$18.8967
Germany	$15.9701
France	$10.2173
United Kingdom	$10.1114
Canada	$6.9007
Brazil	$6.4386
Italy	$5.9980
Australia	$5.1404
South Korea	$4.7033
Spain	$4.5122
Russia	$3.3063
Netherlands	$3.2935
India	$3.0501
Switzerland	$2.9763
Mexico	$2.4863
Sweden	$2.0997
Belgium	$2.0385
Saudi Arabia	$1.6695
Austria	$1.6500
Argentina	$1.5364
Norway	$1.5342
Poland	$1.4560
Iran	$1.4076
Denmark	$1.2535
Turkey	$1.1924
Indonesia	$1.1754
South Africa	$1.1630
Israel	$1.1091
Ireland	$0.9612
Colombia	$0.9534
Chile	$0.9406
Finland	$0.8656
Portugal	$0.7970
New Zealand	$0.7469
Thailand	$0.7388
Czech Republic	$0.6623
United Arab Emirates	$0.6485
Vietnam	$0.6021
Singapore	$0.5949
Philippines	$0.5832
Egypt	$0.5720
Greece	$0.5679
Nigeria	$0.5483
Romania	$0.5145
Malaysia	$0.5127
Peru	$0.4413
Algeria	$0.4007
Cuba	$0.3969
Iraq	$0.3822
Hungary	$0.3747
Ukraine	$0.3177
Pakistan	$0.3114
Ecuador	$0.3101
Bangladesh	$0.2906
Slovakia	$0.2722
Kuwait	$0.2480
Morocco	$0.2344
Qatar	$0.2099
Panama	$0.2061
Uruguay	$0.2009
Kazakhstan	$0.1853
Guatemala	$0.1767
Costa Rica	$0.1669
Kenya	$0.1659
Slovenia	$0.1646
Bulgaria	$0.1634
Serbia	$0.1604
Croatia	$0.1459
Luxembourg	$0.1426
Dominican Republic	$0.1376
Lebanon	$0.1372
Lithuania	$0.1303
Belarus	$0.1297
Jordan	$0.1250
Uzbekistan	$0.1218
Oman	$0.1183
Myanmar	$0.1165
Ethiopia	$0.1151
Venezuela	$0.1141
Turkmenistan	$0.1115
Afghanistan	$0.1094
Sri Lanka	$0.1088
Bolivia	$0.1036
Paraguay	$0.0999
Tanzania	$0.0944
Cyprus	$0.0902
Angola	$0.0886
Ghana	$0.0884
Tunisia	$0.0828
El Salvador	$0.0811
Iceland	$0.0770
Latvia	$0.0758
Ivory Coast	$0.0748
Estonia	$0.0740
DR Congo	$0.0716
Azerbaijan	$0.0700
Honduras	$0.0682
Cambodia	$0.0682
Bosnia and Herzegovina	$0.0611
Sudan	$0.0606
Trinidad and Tobago	$0.0576
Nepal	$0.0573
Cameroon	$0.0561
Uganda	$0.0561
Malta	$0.0551
Armenia	$0.0534
Bahrain	$0.0532
Zambia	$0.0482
Mozambique	$0.0475
Senegal	$0.0410
Georgia	$0.0403
Nicaragua	$0.0400
Namibia	$0.0394
Botswana	$0.0383
Moldova	$0.0342
Jamaica	$0.0339
Burkina Faso	$0.0334
Zimbabwe	$0.0321
North Macedonia	$0.0307
Mauritius	$0.0306
Niger	$0.0295
Bahamas	$0.0277
Mali	$0.0269
Rwanda	$0.0252
Mongolia	$0.0221
Tajikistan	$0.0220
Malawi	$0.0220
Papua New Guinea	$0.0217
Guinea	$0.0214
Madagascar	$0.0202
Haiti	$0.0201
Chad	$0.0198
Laos	$0.0182
Gabon	$0.0177
Montenegro	$0.0161
Maldives	$0.0156
Togo	$0.0155
Kyrgyzstan	$0.0149
Sierra Leone	$0.0138
Equatorial Guinea	$0.0134
Benin	$0.0130
Suriname	$0.0129
Congo	$0.0126
Barbados	$0.0116
Eswatini	$0.0110
Brunei	$0.0107
Mauritania	$0.0102
Lesotho	$0.0099
Liberia	$0.0099
South Sudan	$0.0093
Guyana	$0.0091
Andorra	$0.0086
Burundi	$0.0079
Fiji	$0.0075
Central African Republic	$0.0068
San Marino	$0.0047
Guinea‐Bissau	$0.0046
Belize	$0.0044
Monaco	$0.0039
East Timor	$0.0038
Cape Verde	$0.0036
Eritrea	$0.0033
Saint Lucia	$0.0032
Bhutan	$0.0032
Seychelles	$0.0030
Solomon Islands	$0.0027
Antigua and Barbuda	$0.0026
Djibouti	$0.0025
Comoros	$0.0023
Gambia	$0.0022
Grenada	$0.0022
Saint Kitts and Nevis	$0.0020
Samoa	$0.0019
Micronesia	$0.0017
Palau	$0.0015
Saint Vincent and the Grenadines	$0.0014
Vanuatu	$0.0012
Marshall Islands	$0.0011
Dominica	$0.0011
Tonga	$0.0009
São Tomé and Príncipe	$0.0009
Kiribati	$0.0008
Nauru	$0.0005
Tuvalu	$0.0004
Cook Islands	$0.0004
Niue	$0.0001

Here are how the figures were calculated:

$$\text{EWCE}=\left[\text{PCHCS}\times \mathrm{TP}\right]\times \text{AWCCP},$$

where

*EWCE*—*estimated wound care expenditure (PPP International $)—our estimate of the likely wound care costs*.
*PCHCS*—*per capita health care spend (PPP International $)* ^14^, ^15^—*current published per capita healthcare cost. For the purposes of this editorial, we focused on 2019 to eliminate the bias related to COVID‐19 costs. We will jump ahead to 2022 when they are published*.
*TP is total population*.^16^
*AWCCP*—*average wound care cost percentage* ^1^, ^2^, ^3^, ^4^, ^5^, ^6^, ^7^, ^8^, ^9^, ^10^, ^11^, ^12^—*several published studies have indicated that the percentage of total healthcare costs that is represented by the cost of wounds, ranges from 2% on the low end to 5% on the high end. For the purposes of our calculations, remembering different geographies can be at differing evolutionary stages regarding wound care, we chose the median of 3.5% as the AWCCP*.

While our computation may not be highly scientific, it provides an indication of the likely costs of wounds. These figures can be validated by others locally, but we feel they provide a starting point for those requiring some indication of their costs.

We welcome any input from those involved in such costing studies around the world, perhaps providing a deeper insight. We are happy for others to refine our figures geography by geography providing a more accurate costing where possible.

## CONFLICT OF INTEREST STATEMENT

The authors declare no conflict of interest.

## Data Availability

The data that support the findings of this study are available from the corresponding author upon reasonable request.
